# Patterns of Eye Movements When Observers Judge Female Facial Attractiveness

**DOI:** 10.3389/fpsyg.2017.01909

**Published:** 2017-11-10

**Authors:** Yan Zhang, Xiaoying Wang, Juan Wang, Lili Zhang, Yu Xiang

**Affiliations:** ^1^School of Educational Science, Huazhong University of Science and Technology, Wuhan, China; ^2^Hubei Key Laboratory of Human Development and Mental Health, School of Psychology, Central China Normal University, Wuhan, China

**Keywords:** facial attractiveness, eye movement, attractiveness judgments, fixation patterns, observers, face features

## Abstract

The purpose of the present study is to explore the fixed model for the explicit judgments of attractiveness and infer which features are important to judge the facial attractiveness. Behavioral studies on the perceptual cues for female facial attractiveness implied three potentially important features: averageness, symmetry, and sexual dimorphy. However, these studies did not explained which regions of facial images influence the judgments of attractiveness. Therefore, the present research recorded the eye movements of 24 male participants and 19 female participants as they rated a series of 30 photographs of female facial attractiveness. Results demonstrated the following: (1) Fixation is longer and more frequent on the noses of female faces than on their eyes and mouths (no difference exists between the eyes and the mouth); (2) The average pupil diameter at the nose region is bigger than that at the eyes and mouth (no difference exists between the eyes and the mouth); (3) the number of fixations of male participants was significantly more than female participants. (4) Observers first fixate on the eyes and mouth (no difference exists between the eyes and the mouth) before fixating on the nose area. In general, participants attend predominantly to the nose to form attractiveness judgments. The results of this study add a new dimension to the existing literature on judgment of facial attractiveness. The major contribution of the present study is the finding that the area of the nose is vital in the judgment of facial attractiveness. This finding establish a contribution of partial processing on female facial attractiveness judgments during eye-tracking.

## Introduction

Many researchers, especially psychologists and scientists, have great interest in the human face due to the extremely well-developed ability of humans to recognize, process, and get information from others’ faces for a long time (Little, 2014). Facial attractiveness exerts significant social consequences. Such as, beauty has an impact on upward economic mobility, especially for women (Holmes and Hatch, 1938; Elder, 1969), and attractive people tend to have more dates than less attractive people (Riggio and Woll, 1984). People report that they are more satisfied with their dates when they dated with attractive individuals (Walster et al., 1966; Berscheid et al., 1971). Some women and men admit to being extremely concerned with good looks when looking for potential partners (Buss and Barnes, 1986; Li et al., 2013). In addition, attractiveness can also affect judgments about the seriousness of crimes (Kulka and Kessler, 1978; Mazzella and Feingold, 1994; Little, 2014). In society, attractive people also appear to lead favorable lives and enjoy favorable treatment; attractive individuals pay lower bail (Downs and Lyons, 1991) and are more likely to be hired and promoted for jobs (Marlowe et al., 1996; Chiu and Babcock, 2002) than less attractive individuals, and attractive individuals are more likely to be hired than less attractive ones in interviews (Cash and Kilcullen, 1985).

Some eye-tracking studies asked men to evaluate female faces and bodies to evaluate female overall attractiveness (Nummenmaa et al., 2012). Although both face and body predict women’s overall attractiveness, several studies suggested that women’s faces are better than their bodies as predictors of overall attractiveness (Currie and Little, 2009; Bleske-Rechek et al., 2014). Recently, some neuro-imaging studies found that the reward-related brain area, that is, the orbitofrontal cortex (OFC), is involved in attractiveness perception, with OFC activation being more enhanced for attractive faces than for unattractive faces (Aharon et al., 2001; Winston et al., 2007; Zebrowitz et al., 2009). Additionally, the late positive component (LPC) elicited by attractive faces is larger than that elicited by unattractive faces when subjects are performing an attractiveness rating task (Johnston and Oliver-Rodriguez, 1997; Oliver-Rodríguez et al., 1999).

Studies conducted over the past few years indicated that the key factors in determining facial attractiveness are averageness (Trujillo et al., 2013; Vingilis-Jaremko et al., 2014; Zhang et al., 2014), symmetry (Saxton et al., 2011; Vingilis-Jaremko and Maurer, 2013), sexual dimorphic feature (Perrett et al., 1998; DeBruine et al., 2010), skin health (Jones et al., 2004), and color (Van den Berghe and Frost, 1986). In addition, the list of factors involved in the judgment of facial attractiveness was extended in recent studies. Facial expression (O’Doherty et al., 2003; Golle et al., 2014) and cosmetics use in women (Jones et al., 2014; Ueno et al., 2014) also affect the judgment of facial attractiveness. However, early studies introduced computational models of face recognition which use a whole-face template-like representation (O’Toole et al., 1988; Dailey and Cottrell, 1999). As for face identification, previous studies suggested that the eyes are the most important features for face recognition (Schyns et al., 2002; Hsiao and Cottrell, 2008; Nguyen et al., 2009). On the contrary, a study found that the nose is vital for face identification (Hsiao and Cottrell, 2008). The reason for these differences may be attributed to the different photo materials, participants, and tasks involved in the studies. However, none of these studies used eye movement in the judgment of facial attractiveness and tested which facial regions impacted attractiveness judgment. By tracking an observer’s fixation position, we can directly measure which regions may contribute to the judgment of facial attractiveness.

The traditional view is that “opposites attract, and similarities repel,” that is to say, attraction between opposite sexes is strong, whereas that between the same sexes is weak. Human gender differences in the judgment of facial attractiveness has become popular in recent years (Zhang et al., 2016). Such as some studies found that both sexes will pay more attention to attractive faces than unattractive ones (Aharon et al., 2001; Dai et al., 2010; Hahn and Perrett, 2014). There are proof shows that both men and women pay more attention to heterosexual faces than same-sex faces (Hahn and Perrett, 2014). Some behavioral studies also proved that men and women differ in their attentional adhesion to attractive female faces (Maner et al., 2003, 2007a,b; Zhang et al., 2016). The results of the ERP and behavior indicate that both men and women participants selectively focus on attractive women faces, and different mating-related motives may influence the selective processing of attractive men and women (Maner et al., 2003; Zhang et al., 2016). In another set of studies, Maner et al. (2007a) proved that both genders focus on attractive women but attractive men, therefore posited that these discovery were in line with evolutionary theories which involved adaptive, lower-order mating-related perceptual attunements (Zhang et al., 2016). Moreover, Maner et al. (2007b) used two different experiments to test attentional adhesion to attractive members of the same (potential rivals) and opposite gender (potential mates); the results indicated that attentional adhesion increased in participants who are bisexuality and those who care about threats posed by intersexual competitors (Zhang et al., 2016). A study examined gender differences in recognition memory processing of female facial attractiveness used event-related potentials (ERPs) based on a study-test paradigm. The behavioral data results indicated that both sexes had significantly higher accuracy rates for attractive faces than the unattractive ones, and men reacted faster to unattractive faces. Gender differences on ERPs suggested that attractive faces elicited larger early components such as P1, N170, and P2 in men than in women (Zhang et al., 2016). However, little support is found for eye movement in the judgment of facial attractiveness and tested whether participants of different genders respond differently to facial attractiveness judgment. Besides, all of them cannot explain which regions of stimulus images influence subjects’ judgments. Therefore, we can directly measure that which regions of stimulus images are contribute information to the judgment of facial attractiveness at all times by tracking an observer’s fixation position. In the present study, we presumed that there are differences between gender and fixation patterns during the judgment of facial attractiveness.

## Materials and Methods

### Participants

The participants comprised 43 university students (24 males and 19 females) between 19 and 22 years old. They were all native Chinese Han university students. The research was vetted by an Institutional Review Board. All participants signed an informed consent after they totally understand the procedure, and participants can take “take part in the experiment or not” into account fully. The participants were volunteers and they were paid some small gifts for their participation, such as cap. All of the participants had normal vision, and were right-handed. None of them had neurological or psychiatric disorder.

### Stimuli

Standardized facial stimuli images were developed and validated from a recently published research (Zhang et al., 2011). At first, the study selected 30 images of unfamiliar Chinese young female faces (age range from 20 to 30) with neutral emotional expression among the stimuli validated by Zhang et al. (2011). Then, we recorded the ratings on the attractiveness of the images of 43 participants. There are no significant differences emerged by comparing the attractiveness ratings of the previous 80 participants with the attractiveness ratings of 43 participants in this research [attractive: 5.74, 5.94, *t*(28) = -1.21, *p* = 0.238; unattractive: 2.47,2.58, *t*(28) = -1.04, *p* = 0.307]. There is significant difference between the attractive and unattractive faces in the present research [5.74, 2.47, *t*(28) = 26.08, *p* < 0.001]. Additionally, *t*-test revealed no difference between male and female for attractive and unattractive faces in the present research, [attractive: 5.84, 5.64, *t*(28) = 1.06, *p* = 0.299. unattractive: 2.54,2.41, *t*(28) = 0.97, *p* = 0.341]. Finally, the study selected 30 photographs as the experimental materials (including 15 each with attractive and unattractive faces).

The experimental materials were processed to a uniform size (15 by 15 cm; 450 by 450 pixels), and transformed to 8-bit gray scales in black grounds. In addition, the other physical properties, such as color, luster and lightness, also were standardized. We use Adobe Photoshop to edit the photographs. The evaluated face images of females (including 15 each with high and low attractiveness) were transformed into a unified standard in black and white for avoiding the influence of the complexion of faces on facial attractiveness. In order to ensure the picture showed only the female’s face, we removed the ears, neck and hair, except for the cheek, nose, mouth, and eyes. It should be noted that the faces were not framed in a uniform oval mask, because the faces have different shapes. We have adjusted the mask to suit the face so we can maintain the natural face of the individuals. All the images have front-on view faces with neutral expressions. Photographs were presented in random order under standardized lighting conditions and the background color of the stimuli is black. Individuals in the pictures were unknown to the experimental participants.

### Procedure

The experiment was carried out in a dim light room. Stimuli were presented on a 17-inch EyeLink 1000 eye tracker that with a sampling rate of 1000 Hz can record eye movements. The tracker requires head restraint and participants sat approximately 60–70 cm away from the computer screen, with the horizontal and vertical angles below 6° (Wang et al., 2017). The participants will not be reminded that the equipment are recording their eye movements after the initial calibration. Binocular eye movements were recorded during the judgment task. All the participants knew the experiment procedure before they started that they need to assess the facial attractiveness of the image in the screen one by one. Each trial comprised the following sequence: began with a fixation cross (500 ms), then the target stimulus (20000 ms), and finally followed by a rating screen of infinite time, on which all the participants rated the 30 facial stimuli images attractiveness through input the number from 1 (unattractive) to 9 (attractive). It should be noted that the eye movement screen and rating screen were not in the same screen. The time of rating screen was infinite so that the participants can press keyboard from 1 to 9 when they rated target facial attractiveness. In addition, the effect of the “rating screen of infinite time” is a transition from last seen female face to the next trial, and it was also framed to avoid the eye movement data and behavior data from interfering with each other.

### Statistical Analyses

We used SPSS 17.0 for Windows to analyze the data (SPSS, Inc., Chicago, IL, United States). The repeated-measure ANOVA was performed to compare the gender (male/female) as between factors, and area of interest (nose/mouth/eyes) as within factor. The dependent variables are the index of eye movement, include The time of first fixation, the total fixation time, the number of fixations, and the average pupil diameter. According to the Greenhouse–Geisser method, *p*-values were corrected for deviation from sphericity in all analyses. The section of results showed the main effects and interactions that based on the study hypothesis. If the main effect was significant, we were going to perform Bonferroni *post hoc* test, and simple effect test was conducted if the interaction effect was significant.

## Results

**FIGURE 1:**
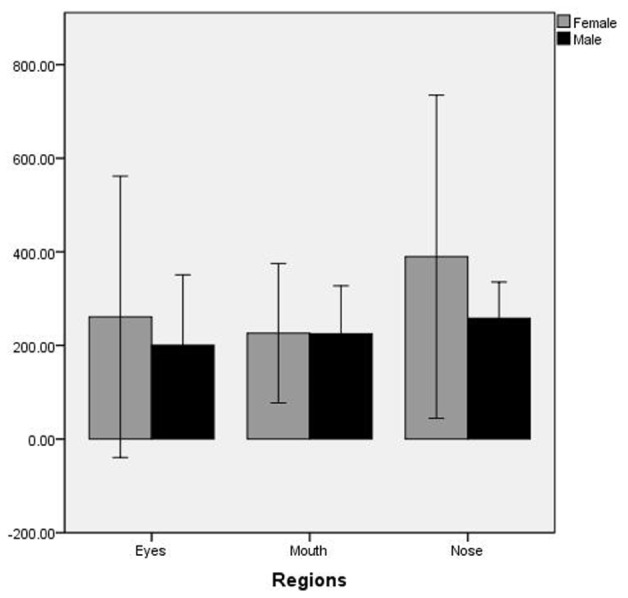
The time of first fixation on regions of interest by male and female participants.

The time of first fixation was analyzed by repeated-measure ANOVA with regions of interest (nose, mouth, and eyes) and gender (male and female) as factors. The main effect of regions of interest is significant, *F*(2,41) = 3.919, *p* = 0.030, η^2^ = 0.09, Observed Power = 0.64. The Bonferroni *post hoc* test revealed that the time of first fixation at nose region was significantly longer than mouth and eyes (*p* < 0.05) (the difference between the mouth and eyes region is not significant). However, the time to first fixation revealed that participants watched the eyes and the mouth first (no significant difference between the eyes and mouth region), followed by the nose. Besides, both the main effect of gender and the interaction of regions of interest and gender were not significant (see **Figure 1**).

**FIGURE 2:**
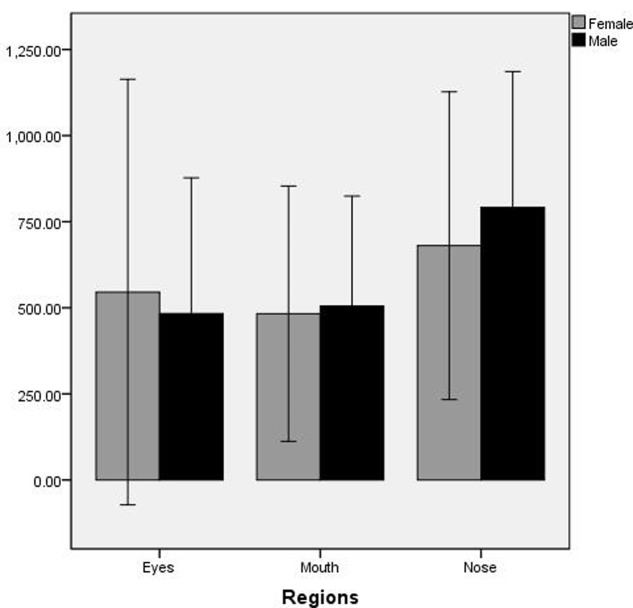
The total fixation time on regions of interest by male and female participants.

The total fixation time was analyzed by repeated-measure ANOVA with regions of interest (nose, mouth, and eyes) and gender (male and female) as factors. There was a significant main effect for the regions of interest, *F*(2,41) = 4.485, *p* = 0.019, η^2^ = 0.10, Observed Power = 0.71. Bonferroni *post hoc* test revealed that the total fixation time at nose region was significantly longer than mouth and eyes (*p* < 0.05) (the difference between the mouth and eyes region is insignificant). However, both the main effect of gender and the interaction of regions of interest and gender were not significant (see **Figure 2**).

**FIGURE 3:**
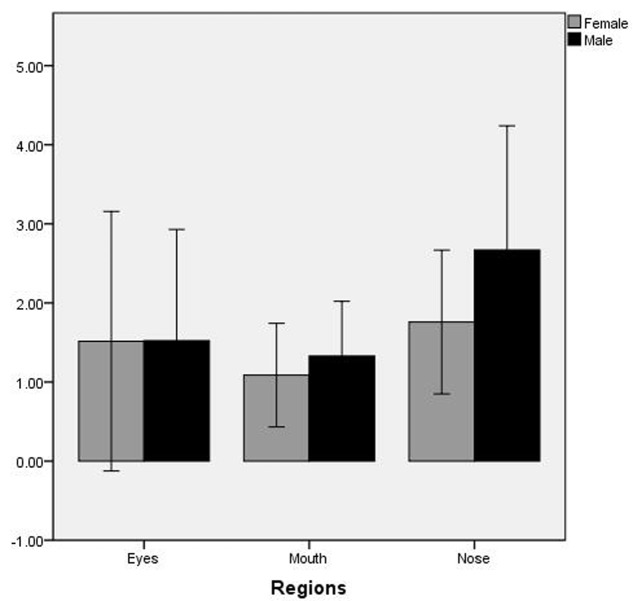
The number of fixations on regions of interest by male and female participants.

The number of fixations was analyzed by repeated-measure ANOVA with regions of interest (nose, mouth, and eyes) and gender (male and female) as factors. The main effect of regions of interest is significant, *F*(2,41) = 8.876, *p* = 0.001, η^2^ = 0.18, Observed Power = 0.96. Bonferroni *post hoc* test revealed that the number of fixations at nose region was significantly more than the eyes and the mouth region (*p* < 0.05) (no significant difference between the mouth and eyes area). The main effect of gender is present, *F*(1,42) = 4.497, *p* = 0.040, η^2^ = 0.10, Observed Power = 0.54. Bonferroni *post hoc* test revealed that the number of fixations of male participants was significantly more than female participants (*p* < 0.05). However, the interaction of regions of interest and gender were not significant (see **Figure 3**).

**FIGURE 4:**
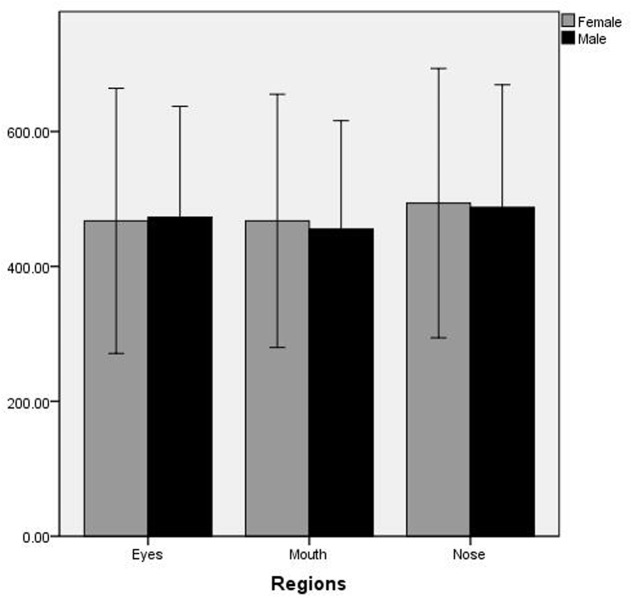
The average pupil diameter on regions of interest for male and female participants.

The average pupil diameter was analyzed by repeated-measure ANOVA with regions of interest (nose, mouth, and eyes) and gender (male and female) as factors. There was a significant main effect for regions of interest, *F*(2,41) = 13.838, *p* < 0.001, η^2^= 0.25, Observed Power = 0.99. Bonferroni *post hoc* test revealed that the average pupil diameter at nose region was significantly bigger than the eyes and the mouth region (*p* < 0.05) (no significant difference between the mouth and eyes area). However, both the main effect of gender and the interaction of regions of interest and gender were not significant (see **Figure 4**).

## Discussion

Our results suggest that the first and total fixation time for the nose region is significantly longer than that for the eyes and mouth region (*p* < 0.05) and that the number of fixations on the nose region is significantly greater than the eyes and mouth (*p* < 0.05). In addition, there has no significant difference between the mouth and eyes in terms of the indexes of fixations. These findings are in line with existing research (Hsiao and Cottrell, 2008). Hsiao and Cottrell (2008) noted that two fixations are sufficient when people recognize face; the position of the two fixations are around the center, and the first fixation located to the left of the center slightly.

Empirical evidence of this study revealed that the regions of interest on the nose differed significantly from eyes and mouth for the average pupil diameter, and there has no significant difference between the mouth and eyes in the average pupil diameter. Some past research discovered that pupil diameter increases as a result of sexual arousal (Bernick et al., 1971; Hamel, 1974) and novelty (Aboyoun and Dabbs, 1998). A recent study found a positive correlation between pupil size and aesthetic ratings, which means that the bigger the pupil size, the higher the aesthetic rank are (Blackburn and Schirillo, 2012; Pain and Central Nervous System Week, 2012). These results suggested that the nose is the most important component for the judgment of facial attractiveness. In addition, luminance can vary across images and thus causes considerable variation in pupil size (Loewenfeld and Lowenstein, 1993; Bradley et al., 2008). Therefore, photographs are developed under standardized lighting conditions to reduce this bias.

However, only significant effect was observed for gender on the number of fixations, and male participants was significantly more than female participants (*p* < 0.05). No significant regions of interest × gender interaction were identified in any index of eye movement in the present study. We presumed that males are pay more attention to females’ facial attractiveness. This view is consistent with the evolutionary perspectives because only through this can they make mate choices and produce offspring. These results are consistent with existing research suggesting a gender difference in the judgment of facial attractiveness (Kniffin and Wilson, 2004; Yamamoto et al., 2009). The study by Yamamoto et al. (2009) found that excessive motivation to prolong men’s viewing time of normal babies vs. shorten women’s exposure to abnormal babies. The study of Kniffin and Wilson (2004) showed that the non-physical factors have more impact on females than males, and there has large individual differences in sex. This finding is also consistent with the traditional view and the evolutionary point of view. The traditional view is that “opposites attract, and similarities repel,” that is to say, attraction between opposite sexes is strong, whereas that between the same sexes is weak.

In addition, our study revealed that participants watch the eyes and mouth first, followed by the nose, and no significant difference was observed between the mouth and the eyes; such finding is in line with existing research (Vinette et al., 2004; Nguyen et al., 2009). The study by Vinette et al. (2004) noted that the eyes have spatio-temporal dynamics that help face recognition in a flash. Nguyen’s study (Nguyen et al., 2009) added that age and fatigue judgments are related to preferential attention toward the eye region. Another similar investigation also found that personal evaluation was moderated by the direction of gaze shifts (Mason et al., 2005). From the perspective of face identification, numerous studies generated different conclusions. Some studies indicated that the eyes are the most important features for face identification (Schyns et al., 2002; Hsiao and Cottrell, 2008; Nguyen et al., 2009) on the contrary, another study suggested that nose is vital for face identification (Hsiao and Cottrell, 2008). One possible reason for these differences could be the use of different photo materials, participants, and tasks.

These findings suggest that the nose might be vital in the judgment of facial attractiveness. Based on the saliency literature (Santangelo, 2015), visual saliency have been checked to indicate that nose regions are not more salient than the other regions of face from both the high attractive faces and the low attractive faces (see the figures in the Supplementary Material). Some study suggested that nose is vital for face identification (Hsiao and Cottrell, 2008). East Asians recognize faces by focusing on the region in which integrating information holistically would be optimal and economical, that is, the center of the face (i.e., nose). Because retinal cell density differ among areas in eyes and visual resolution decrease sharply toward the peripheral visual field, the center of the face may become the most favorable spatial location to obtain facial feature information. An early empirical work discovered that direct or excessive eye contact may be considered rude in East Asian cultures (Argyle and Cook, 1976) and that this social norm is probably the cause of gaze avoidance among East Asian observers. To some extent, the holistic perceptual strategies used by East Asian observers could explain the East Asian fixation bias toward the nose region (Chua et al., 2005; Miyamoto et al., 2006). However, the previous studies in the bubbles process suggested that the most important features in face identification are the eyes (Schyns et al., 2002; Nguyen et al., 2009). The standard measure to modeling eye fixation and visual attention are usually based on a salient map, which is calculated according to biological motivated features selection for information maximization. These models predict that the observer gaze the eyes when they view faces, but this is not consistent with our results that suggested eye movements in the facial attractiveness judgment task are different from those in the scene viewing task or visual search task (Yamada and Cottrell, 1995; Itti et al., 1998).

In sum, the results of this study add a new dimension to the existing literature on judgment of facial attractiveness. The major contribution of the present study is the finding that the area of the nose is vital in the judgment of facial attractiveness. This finding establish a contribution of partial processing on female facial attractiveness judgments during eye-tracking. Considering these novel findings, we believe that future work should also use eye movement to explore facial attractiveness along with other factors, such as social status, personality, and facial emotion. Nevertheless, additional research is necessary to assess the ability of observers to rate male or female objects.

## Ethics Statement

Each participant signed an informed consent before the procedure was fully explained. Approval was granted by the Research Ethics Committee of the School of Educational Sciences, Huazhong University of Science and Technology, China. All participants were right-handed and had normal vision, with no self-reported history of neurological or psychiatric disorder. All the authors have approved the manuscript and agree with submission to your esteemed journal.

## Author Contributions

Conceived and designed the experiments: YZ. Recruitment and payment of participants: YZ. Performed the experiments: YZ. Analyzed the data: YZ, LZ, XW, and JW. Wrote and revised the paper: LZ, YZ, XW, JW, and YX.

## Conflict of Interest Statement

The authors declare that the research was conducted in the absence of any commercial or financial relationships that could be construed as a potential conflict of interest. The reviewer FK declared a shared affiliation, though no other collaboration, with one of the authors YZ to the handling Editor.

## References

[B1] Aboyoun D. C. Dabbs J. M. (1998). The Hess pupil dilation findings: sex or novelty? *Soc. Behav. Pers.* 26 415–419. 10.2224/sbp.1998.26.4.415

[B2] Aharon I. Etcoff N. Ariely D. Chabris C. F. O’Connor E. Breiter H. C. (2001). Beautiful faces have variable reward value: fMRI and behavioral evidence. *Neuron* 32 537–551. 10.1016/S0896-6273(01)00491-3 11709163

[B3] Argyle M. Cook M. (1976). *Gaze and Mutual Gaze.* Oxford: Cambridge University Press, 210.

[B4] Bernick N. Kling A. Borowitz G. (1971). Physiologic differentiation of sexual arousal and anxiety. *Psychosom. Med.* 33 341–352. 10.1097/00006842-197107000-00004 5112331

[B5] Berscheid E. Dion K. Walster E. Walster G. W. (1971). Physical attractiveness and dating choice: a test of the matching hypothesis. *J. Exp. Soc. Psychol.* 7 173–189. 10.1016/0022-1031(71)90065-5

[B6] Blackburn K. Schirillo J. (2012). Emotive hemispheric differences measured in real-life portraits using pupil diameter and subjective aesthetic preferences. *Exp. Brain Res.* 219 447–455. 10.1007/s00221-012-3091-y 22526951

[B7] Bleske-Rechek A. Kolb C. M. Stern A. S. Quigley K. Nelson L. A. (2014). Face and body: independent predictors of women’s attractiveness. *Arch. Sex. Behav.* 43 1355–1365. 10.1007/s10508-014-0304-4 24830907

[B8] Bradley M. M. Miccoli L. Escrig M. A. Lang P. J. (2008). The pupil as a measure of emotional arousal and autonomic activation. *Psychophysiology* 45 602–607. 10.1111/j.1469-8986.2008.00654.x 18282202 PMC3612940

[B9] Buss D. M. Barnes M. (1986). Preferences in human mate selection. *J. Pers. Soc. Psychol.* 50 559–570. 10.1037/0022-3514.50.3.559

[B10] Cash T. F. Kilcullen R. N. (1985). The aye of the beholder: susceptibility to sexism and beautyism in the evaluation of managerial applicants1. *J. Appl. Soc. Psychol.* 15 591–605. 10.1111/j.1559-1816.1985.tb00903.x

[B11] Chiu R. K. Babcock R. D. (2002). The relative importance of facial attractiveness and gender in Hong Kong selection decisions. *Int. J. Hum. Resour. Manag.* 13 141–155. 10.1080/09585190110092857

[B12] Chua H. F. Boland J. E. Nisbett R. E. (2005). Cultural variation in eye movements during scene perception. *Proc. Natl. Acad. Sci. U.S.A.* 102 12629–12633. 10.1073/pnas.0506162102 16116075 PMC1194960

[B13] Currie T. E. Little A. C. (2009). The relative importance of the face and body in judgments of human physical attractiveness. *Evol. Hum. Behav.* 30 409–416. 10.1016/j.evolhumbehav.2009.06.005

[B14] Dai X. Brendl C. M. Ariely D. (2010). Wanting, liking, and preference construction. *Emotion* 10 324–34. 10.1037/a0017987 20515222

[B15] Dailey M. N. Cottrell G. W. (1999). Organization of face and object recognition in modular neural network models. *Neural Netw.* 12 1053–1074. 10.1016/S0893-6080(99)00050-7 12662645

[B16] DeBruine L. M. Jones B. C. Smith F. G. Little A. C. (2010). Are attractive men’s faces masculine or feminine? The importance of controlling confounds in face stimuli. *J. Exp. Psychol. Hum. Percept. Perform.* 36 751–758. 10.1037/a0016457 20515201

[B17] Downs A. C. Lyons P. M. (1991). Natural observations of the links between attractiveness and initial legal judgments. *Pers. Soc. Psychol. Bull.* 17 541–547. 10.1177/0146167291175009

[B18] Elder G. H. Jr. (1969). Appearance and education in marriage mobility. *Am. Sociol. Rev.* 34 519–533. 10.2307/2091961 5811582

[B19] Golle J. Mast F. W. Lobmaier J. S. (2014). Something to smile about: the interrelationship between attractiveness and emotional expression. *Cogn. Emot.* 28 298–310. 10.1080/02699931.2013.817383 23875865

[B20] Hahn A. C. Perrett D. I. (2014). Neural and behavioral responses to attractiveness in adult and infant faces. *Neurosci. Biobehav. Rev.* 46 591–603. 10.1016/j.neubiorev.2014.08.015 25199981

[B21] Hamel R. F. (1974). Female subjective and pupillary reaction to nude male and female figures. *J. Psychol.* 87 171–175. 10.1080/00223980.1974.9915687 4443952

[B22] Holmes S. Hatch C. (1938). Personal appearance as related to scholastic records and marriage selection in college women. *Hum. Biol.* 10 65–76.

[B23] Hsiao J. H.-W. Cottrell G. (2008). Two fixations suffice in face recognition. *Psychol. Sci.* 19 998–1006. 10.1111/j.1467-9280.2008.02191.x 19000210 PMC7360057

[B24] Itti L. Koch C. Niebur E. (1998). A model of saliency-based visual attention for rapid scene analysis. *IEEE Trans. Pattern Anal. Mach. Intell.* 20 1254–1259. 10.1109/34.730558 17688904

[B25] Johnston V. S. Oliver-Rodriguez J. C. (1997). Facial beauty and the late positive component of event-related potentials. *J. Sex Res.* 34 188–198. 10.1080/00224499709551884

[B26] Jones A. L. Kramer R. S. Ward R. (2014). Miscalibrations in judgements of attractiveness with cosmetics. *Q. J. Exp. Psychol.* 67 2060–2068. 10.1080/17470218.2014.908932 24670156

[B27] Jones B. C. Little A. C. Burt D. M. Perrett D. I. (2004). When facial attractiveness is only skin deep. *Perception* 33 569–576. 10.1068/p3463 15250662

[B28] Kniffin K. M. Wilson D. S. (2004). The effect of nonphysical traits on the perception of physical attractiveness: three naturalistic studies. *Evol. Hum. Behav.* 25 88–101. 10.1016/S1090-5138(04)00006-6

[B29] Kulka R. A. Kessler J. B. (1978). Is justice really blind? –The influence of litigant physical attractiveness on juridical judgment1. *J. Appl. Soc. Psychol.* 8 366–381. 10.1111/j.1559-1816.1978.tb00790.x

[B30] Li N. P. Yong J. C. Tov W. Sng O. Fletcher G. J. Valentine K. A. (2013). Mate preferences do predict attraction and choices in the early stages of mate selection. *J. Pers. Soc. Psychol.* 105 757–776. 10.1037/a0033777 23915041

[B31] Little A. C. (2014). Facial attractiveness. *Wiley Interdiscip. Rev. Cogn. Sci.* 5 621–634. 10.1002/wcs.1316 26308869

[B32] Loewenfeld I. E. Lowenstein O. (1993). *The Pupil: Anatomy, Physiology, and Clinical Applications.* Ames, IA: Iowa State University Press.

[B33] Maner J. K. Gailliot M. T. DeWall C. N. (2007a). Adaptive attentional attunement: evidence for mating-related perceptual bias. *Evol. Hum. Behav.* 28 28–36. 10.1016/j.evolhumbehav.2006.05.006

[B34] Maner J. K. Gailliot M. T. Rouby D. A. Miller S. L. (2007b). Can’t take my eyes off you: attentional adhesion to mates and rivals. *J. Pers. Soc. Psychol.* 93 389–401. 17723055 10.1037/0022-3514.93.3.389

[B35] Maner J. K. Kenrick D. T. Becker D. V. Delton A. W. Hofer B. Wilbur C. J. (2003). Sexually selective cognition: beauty captures the mind of the beholder. *J. Pers. Soc. Psychol.* 85 1107–1120. 10.1037/0022-3514.85.6.1107 14674817

[B36] Marlowe C. M. Schneider S. L. Nelson C. E. (1996). Gender and attractiveness biases in hiring decisions: are more experienced managers less biased? *J. Appl. Psychol.* 81 11–21. 10.1037/0021-9010.81.1.11

[B37] Mason M. F. Tatkow E. P. Macrae C. N. (2005). The look of love gaze shifts and person perception. *Psychol. Sci.* 16 236–239. 10.1111/j.0956-7976.2005.00809.x 15733205

[B38] Mazzella R. Feingold A. (1994). The effects of physical attractiveness, race, socioeconomic status, and gender of defendants and victims on judgments of mock jurors: a meta-analysis1. *J. Appl. Soc. Psychol.* 24 1315–1338. 10.1111/j.1559-1816.1994.tb01552.x

[B39] Miyamoto Y. Nisbett R. E. Masuda T. (2006). Culture and the physical environment holistic versus analytic perceptual affordances. *Psychol. Sci.* 17 113–119. 10.1111/j.1467-9280.2006.01673.x 16466418

[B40] Nguyen H. T. Isaacowitz D. M. Rubin P. A. (2009). Age-and fatigue-related markers of human faces: an eye-tracking study. *Ophthalmology* 116 355–360. 10.1016/j.ophtha.2008.10.007 19084276

[B41] Nummenmaa L. Hietanen J. K. Santtila P. Hyona J. (2012). Gender and visibility of sexual cues influence eye movements while viewing faces and bodies. *Arch. Sex. Behav.* 41 1439–1451. 10.1007/s10508-012-9911-0 22402995

[B42] O’Doherty J. Winston J. Critchley H. Perrett D. Burt D. M. Dolan R. J. (2003). Beauty in a smile: the role of medial orbitofrontal cortex in facial attractiveness. *Neuropsychologia* 41 147–155. 10.1016/S0028-3932(02)00145-8 12459213

[B43] Oliver-Rodríguez J. C. Guan Z. Johnston V. S. (1999). Gender differences in late positive components evoked by human faces. *Psychophysiology* 36 176–185. 10.1111/1469-8986.3620176 10194964

[B44] O’Toole A. J. Millward R. B. Anderson J. A. (1988). A physical system approach to recognition memory for spatially transformed faces. *Neural Netw.* 1 179–199. 10.1016/0893-6080(88)90025-1

[B45] Pain and Central Nervous System Week (2012). *Studies from Wake Forest University in the Area of Central Nervous System Described.* Bethesda MD: Pain and Central Nervous System Week, 572.

[B46] Perrett D. Lee K. Penton-Voak I. Rowland D. Yoshikawa S. Burt D. (1998). Effects of sexual dimorphism on facial attractiveness. *Nature* 394 884–887. 10.1038/29772 9732869

[B47] Riggio R. E. Woll S. B. (1984). The role of nonverbal cues and physical attractiveness in the selection of dating partners. *J. Soc. Pers. Relationsh.* 1 347–357. 10.1177/0265407584013007

[B48] Santangelo V. (2015). Forced to remember: when memory is biased by salient information. *Behav. Brain Res.* 283 1–10. 10.1016/j.bbr.2015.01.013 25595422

[B49] Saxton T. K. Debruine L. M. Jones B. C. Little A. C. Craig Roberts S. (2011). A longitudinal study of adolescents’ judgments of the attractiveness of facial symmetry, averageness and sexual dimorphism. *J. Evol. Psychol.* 9 43–55. 10.1556/JEP.9.2011.22.1

[B50] Schyns P. G. Bonnar L. Gosselin F. (2002). Show me the features! understanding recognition from the use of visual information. *Psychol. Sci.* 13 402–409. 10.1111/1467-9280.00472 12219805

[B51] Trujillo L. T. Jankowitsch J. M. Langlois J. H. (2013). Beauty is in the ease of the beholding: a neurophysiological test of the averageness theory of facial attractiveness. *Cogn. Affect. Behav. Neurosci.* 14 1061–1076. 10.3758/s13415-013-0230-2 24326966 PMC4053512

[B52] Ueno A. Ito A. Kawasaki I. Kawachi Y. Yoshida K. Murakami Y. (2014). Neural activity associated with enhanced facial attractiveness by cosmetics use. *Neurosci. Lett.* 566 142–146. 10.1016/j.neulet.2014.02.047 24598437

[B53] Van den Berghe P. L. Frost P. (1986). Skin color preference, sexual dimorphism and sexual selection: a case of gene culture co-evolution?^∗^. *Ethn. Racial Stud.* 9 87–113. 10.1080/01419870.1986.9993516

[B54] Vinette C. Gosselin F. Schyns P. G. (2004). Spatio-temporal dynamics of face recognition in a flash: it’s in the eyes. *Cogn. Sci.* 28 289–301. 10.1207/s15516709cog2802_8

[B55] Vingilis-Jaremko L. Maurer D. (2013). The influence of symmetry on children’s judgments of facial attractiveness. *Perception* 42 302–320. 10.1068/p7371 23837207

[B56] Vingilis-Jaremko L. Maurer D. Gao X. (2014). The influence of averageness on judgments of facial attractiveness: no own-age or own-sex advantage among children attending single-sex schools. *J. Exp. Child Psychol.* 120 1–16. 10.1016/j.jecp.2013.10.006 24326246

[B57] Walster E. Aronson V. Abrahams D. Rottman L. (1966). Importance of physical attractiveness in dating behavior. *J. Pers. Soc. Psychol.* 4 508–516. 10.1037/h0021188 6008393

[B58] Wang Y. Gu R. Luo Y. J. Zhou C. (2017). The interaction between state and dispositional emotions in decision making: an ERP study. *Biol. Psychol.* 123 126–135. 10.1016/j.biopsycho.2016.11.009 27887980

[B59] Winston J. S. O’Doherty J. Kilner J. M. Perrett D. I. Dolan R. J. (2007). Brain systems for assessing facial attractiveness. *Neuropsychologia* 45 195–206. 10.1016/j.neuropsychologia.2006.05.009 16828125

[B60] Yamada K. Cottrell G. W. (1995). “A model of scan paths applied to face recognition,” in *Proceedings of the 17th Annual Conference of the Cognitive Science Society* (Mahwah, NJ: Lawrence Erlbaum), 55–60.

[B61] Yamamoto R. Ariely D. Chi W. Langleben D. D. Elman I. (2009). Gender differences in the motivational processing of babies are determined by their facial attractiveness. *PLOS ONE* 4:e6042. 10.1371/journal.pone.0006042 19554100 PMC2698285

[B62] Zebrowitz L. A. Luevano V. X. Bronstad P. M. Aharon I. (2009). Neural activation to babyfaced men matches activation to babies. *Soc. Neurosci.* 4 1–10. 10.1080/17470910701676236 19101842 PMC4037367

[B63] Zhang Y. Kong F. Chen H. Jackson T. Han L. Meng J. (2011). Identifying cognitive preferences for attractive female faces: an event-related potential experiment using a study-test paradigm. *J. Neurosci. Res.* 89 1887–1893. 10.1002/jnr.22724 21805493

[B64] Zhang Y. Kong F. Zhong Y. Kou H. (2014). Personality manipulations: do they modulate facial attractiveness ratings? *Pers. Individ. Dif.* 70 80–84. 10.1016/j.paid.2014.06.033

[B65] Zhang Y. Wei B. Zhao P. Zheng M. Zhang L. (2016). Gender differences in memory processing of female facial attractiveness: evidence from event-related potentials. *Neurocase* 22 317–323. 10.1080/13554794.2016.1151532 26928269

