# Supplementary figures and images for: Patterns of Eye Movements When Observers Judge Female Facial Attractiveness
Source: Front Psychol. 2017 Nov 10;8:1909. doi: 10.3389/fpsyg.2017.01909 (PMC5701615; doi:10.3389/fpsyg.2017.01909)

High attractive:

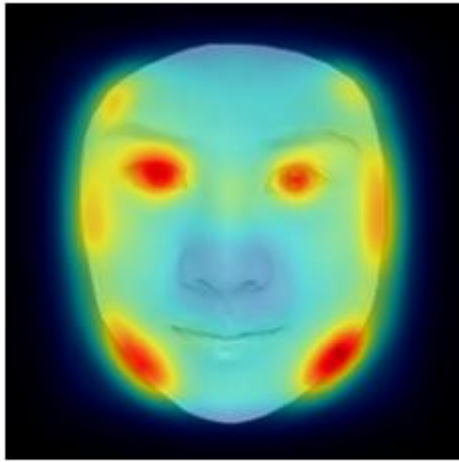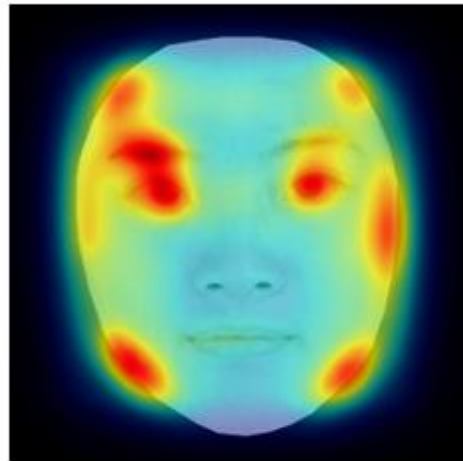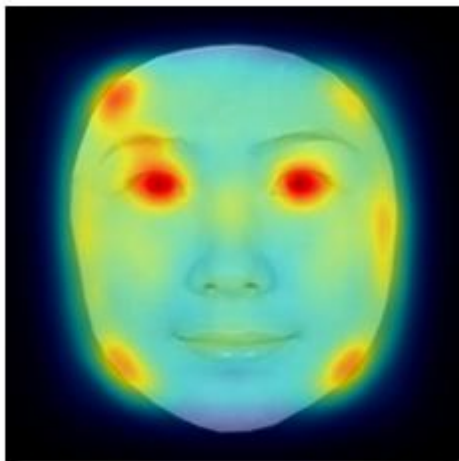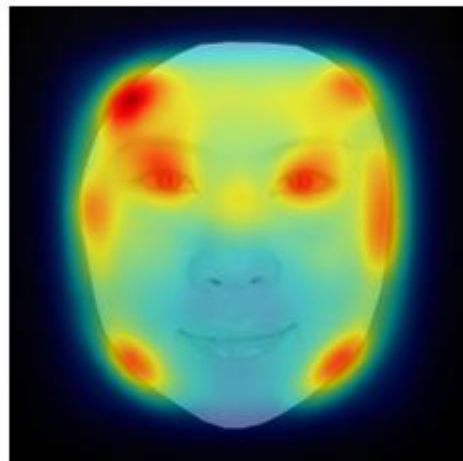

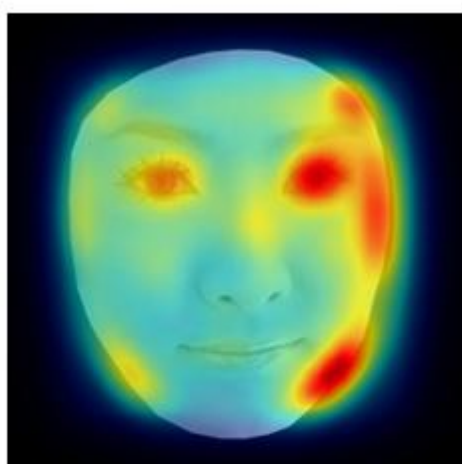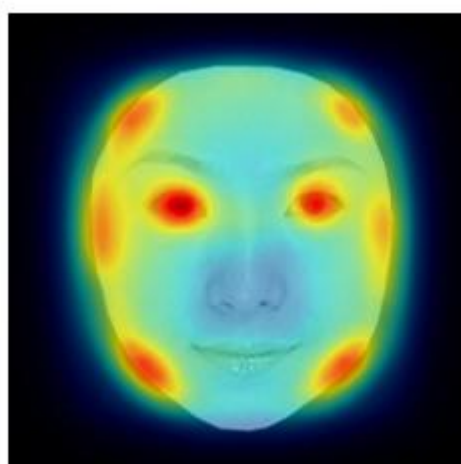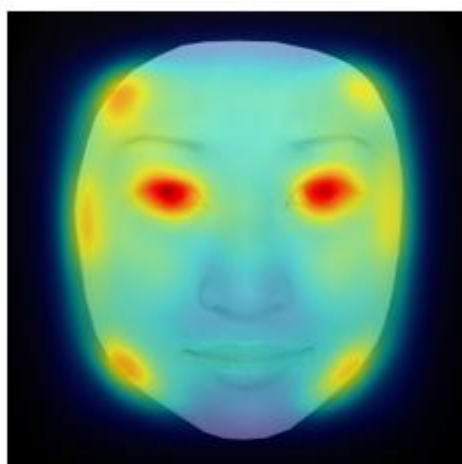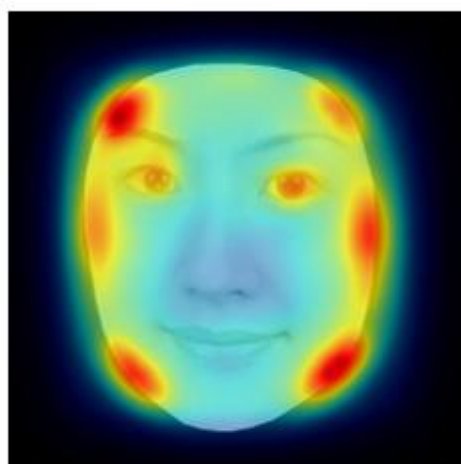

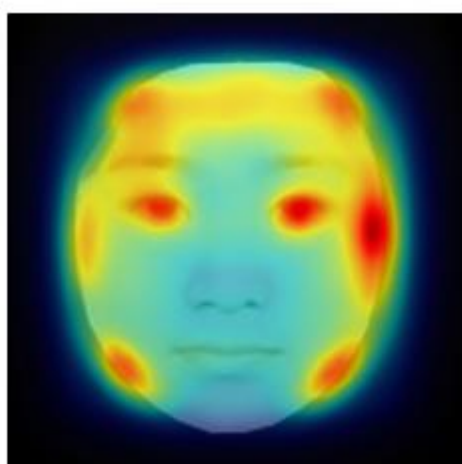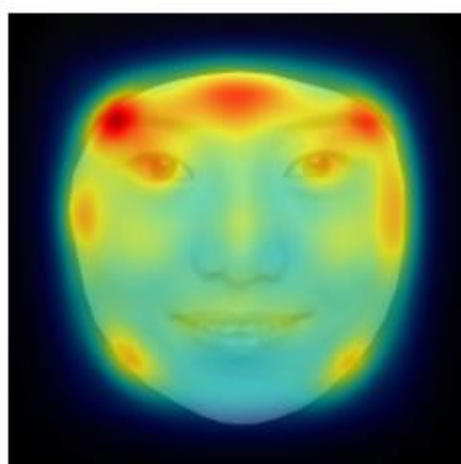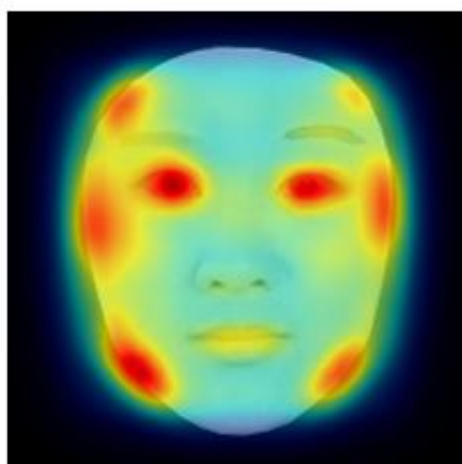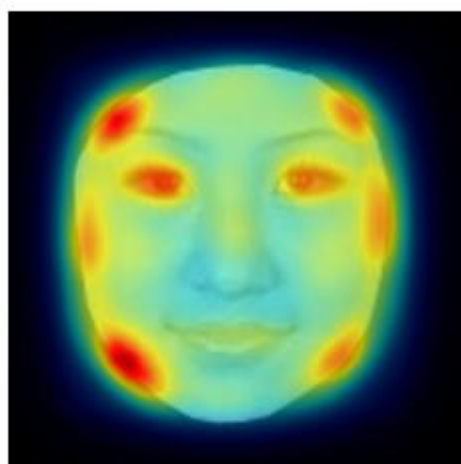

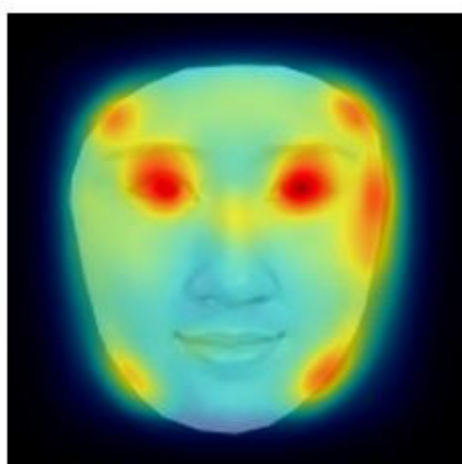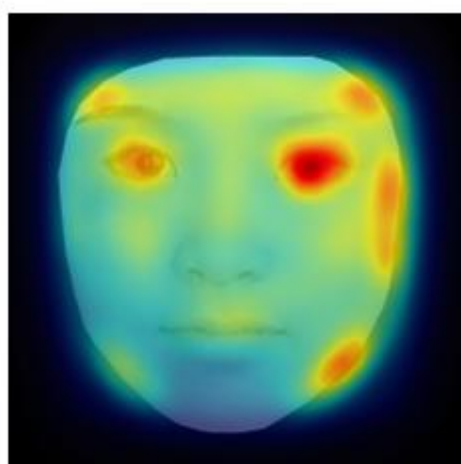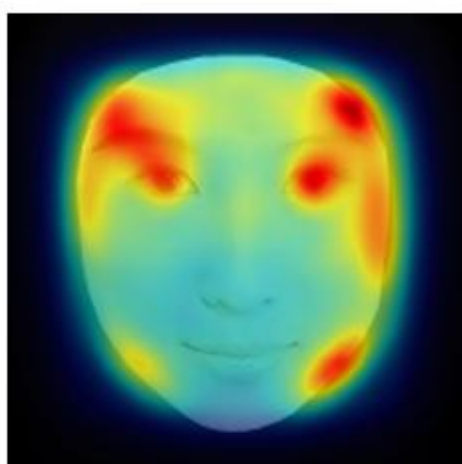

Low attractive:

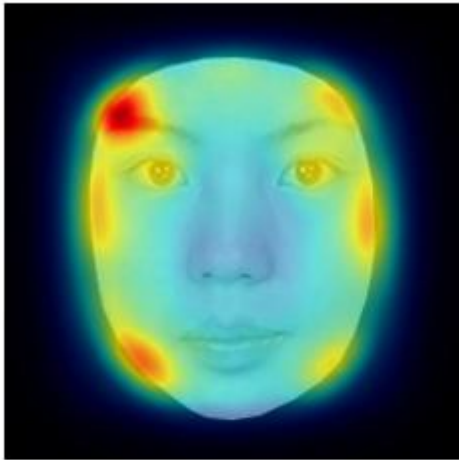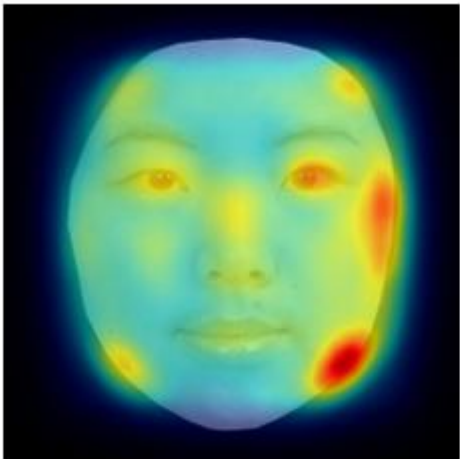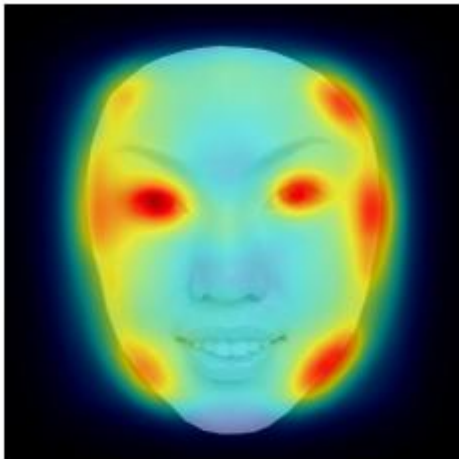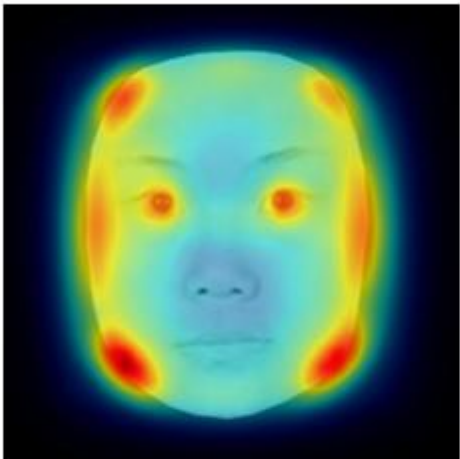

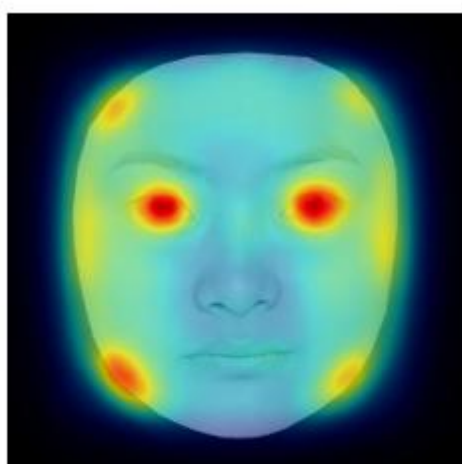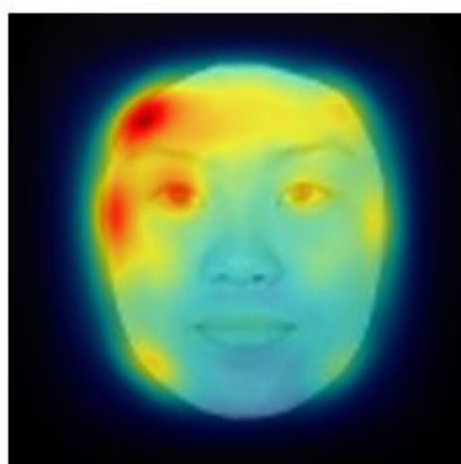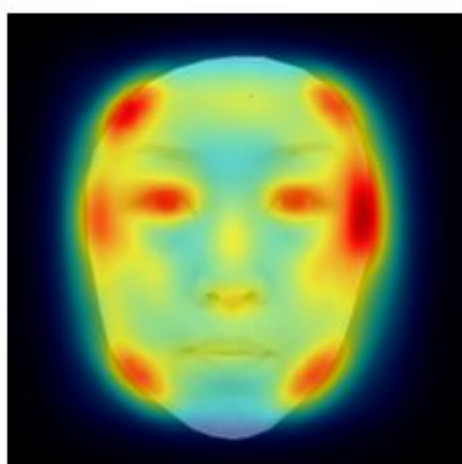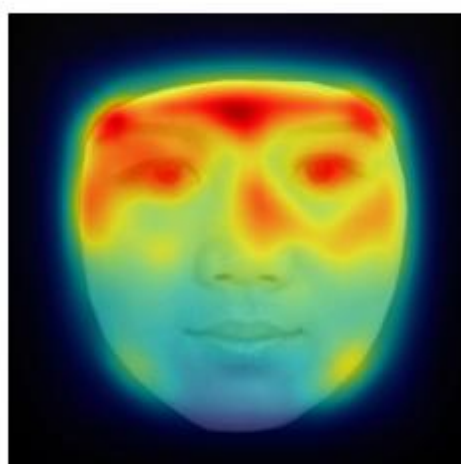

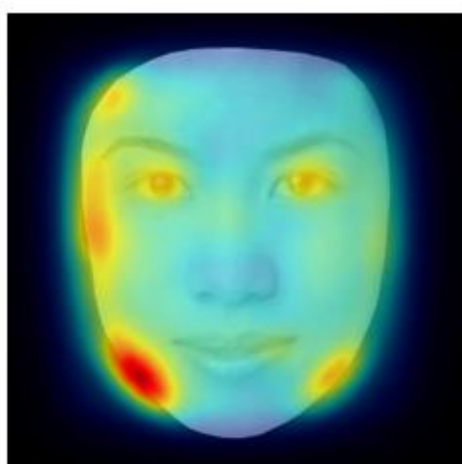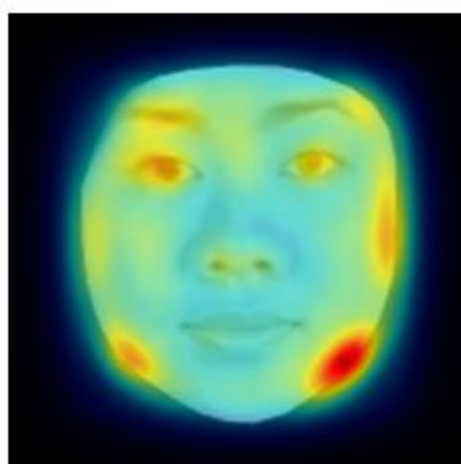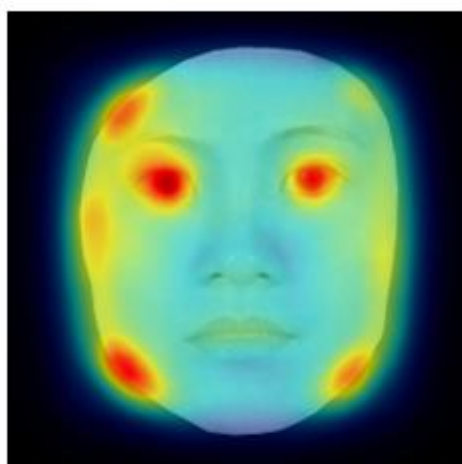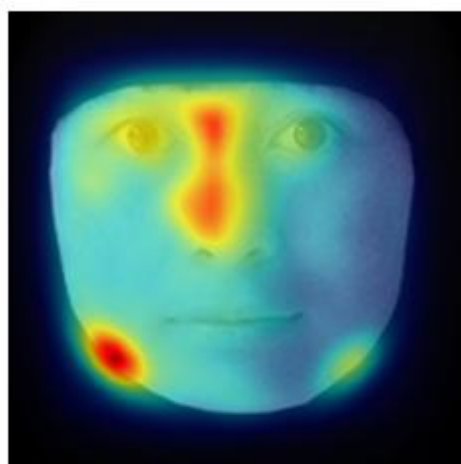

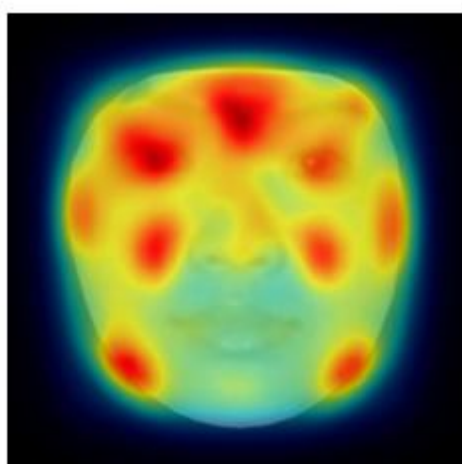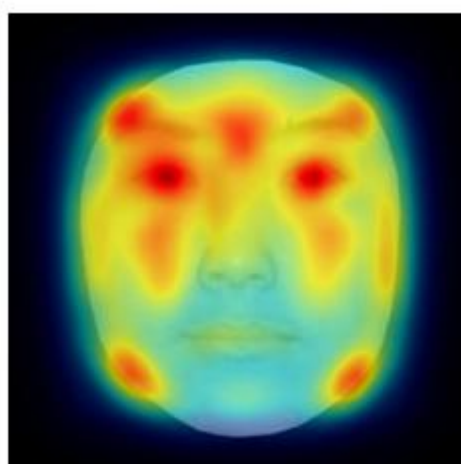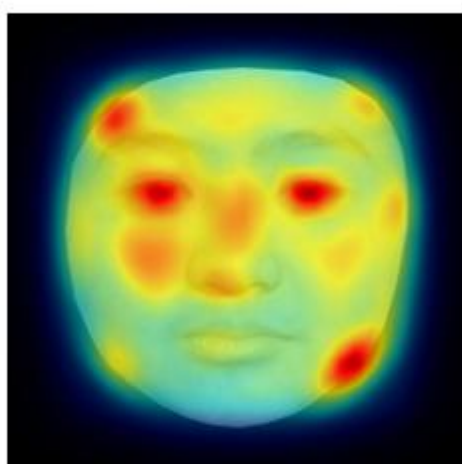

Supplement: Supplementary file 2 [file Presentation_2.PDF]
