# Supplementary material for: Patterns of Eye Movements When Observers Judge Female Facial Attractiveness
Source: Front Psychol. 2017 Nov 10;8:1909. doi: 10.3389/fpsyg.2017.01909 (PMC5701615; doi:10.3389/fpsyg.2017.01909)
Supplement: Supplementary file 1 [file Presentation_1.PDF]

Dear editor and referee,

Thank you for providing the opportunity to resubmit a revision in accordance with the referees' recommendations. We appreciate the referees’ enthusiasm and thoughtful comments regarding the initial draft. Although it has taken time for us to digest and address these comments, all of the reviewer suggestions and comments have been incorporated into the attached revision.

The referees’ comments are presented below followed by our response and details for locating changes to the paper. Thank you in advance for the time and care you will have devoted to this revision.

Thank you again for providing **Frontiers in Psychology, section Cognitive Science** as a potential outlet for this work.

Cordially,

The authors

Patterns of Eye Movements when Male and Female Observers Judge Female Attractiveness

Comments from the Editors and Reviewers:

Reviewer 3 |

I really appreciate the efforts made to improve this revised version of the manuscript. However, my major point still need to be successfully addressed:

The literature on visual saliency has been misinterpret here. Indeed the previous literature found that saliency models are predictive of fixations patterns during free visual exploration, just like in the current task, in which participants are required to freely explore female faces. The reduction of saliency predictivity is instead related to visual search tasks, in which a specific target has to be searched for in a given scene.

Given that the main finding of this study is somewhat surprising (i.e., it seems to be the nose instead of the eyes or the mouth to be the most relevant feature to judge attractiveness) I think is really necessary to check whether “nose” regions are more salient than the other regions of face. Then, I still believe that to check the effect of visual saliency is extremely important to confirm the current findings (and it is also easy to perform using free softwares, as the one I indicated before).

If for any reason the authors cannot do this important check they should at the very list make explicit in the manuscript why to check the impact of saliency might be relevant here (based on the “correct” interpretation of Santangelo 2015 and the saliency literature, please see above) and that future studies need to carefully address such a potential confound.

Without this I anticipate that I will not endorse the publication of the current manuscript.

Response: Thank you very much for giving these comments. At first, we are sorry for misunderstand the literature on visual saliency. Recently, we have re-read the

Santangelo (2015) study seriously, and you are right. So, we have check whether “nose” regions are more salient than the other regions of face with the software you indicated. We have already installed the MATLAB software, and download the gbvs.zip file to compute a saliency map and display a saliency map on top of an image. The example code as follows:

```
命令行窗口
>> run('D:\系统\GBVS\gbvs\gbvs\gbvs_install.m')
>> img = imread('12.bmp');
>> map = gbvs(img);
>> show_imgnmap( img , map );
fx >>
```

And, the saliency map of all 30 images have added in the MATLAB image file. Finally, we find that “nose” regions are not more salient than the other regions of face. In the group of high attractive, we can see that “eyes” regions and part of cheek regions are more salient but not “nose” regions. (as follows)

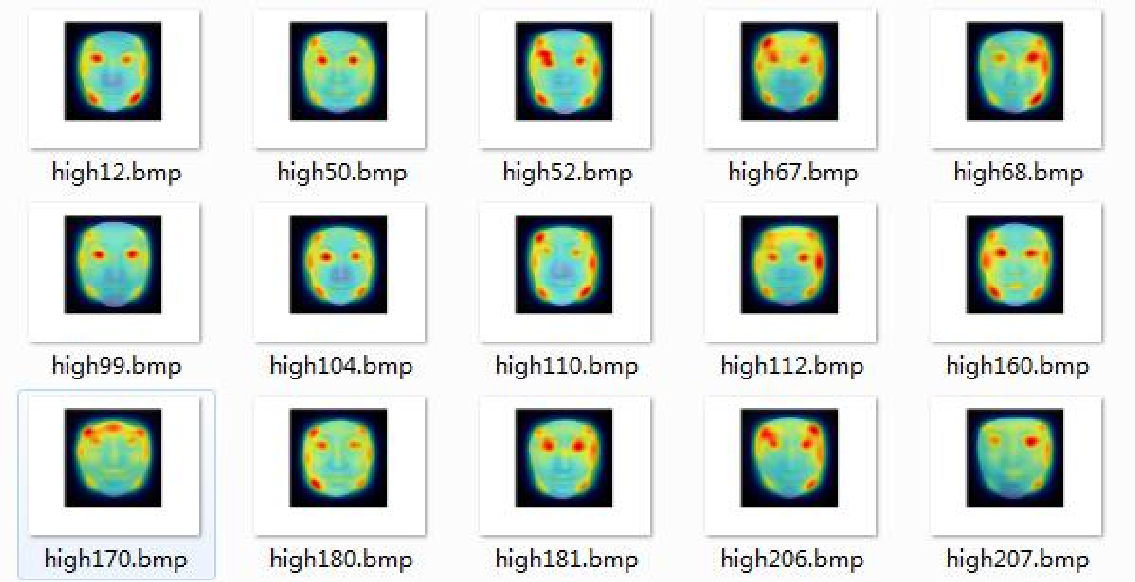

But in the group of low attractive, we think there do not have regular. There are many of salient regions. In some images, we can see that “eyes” regions are more salient. But in other images, the forehead region or the cheek region or both are salient regions. (as follows)

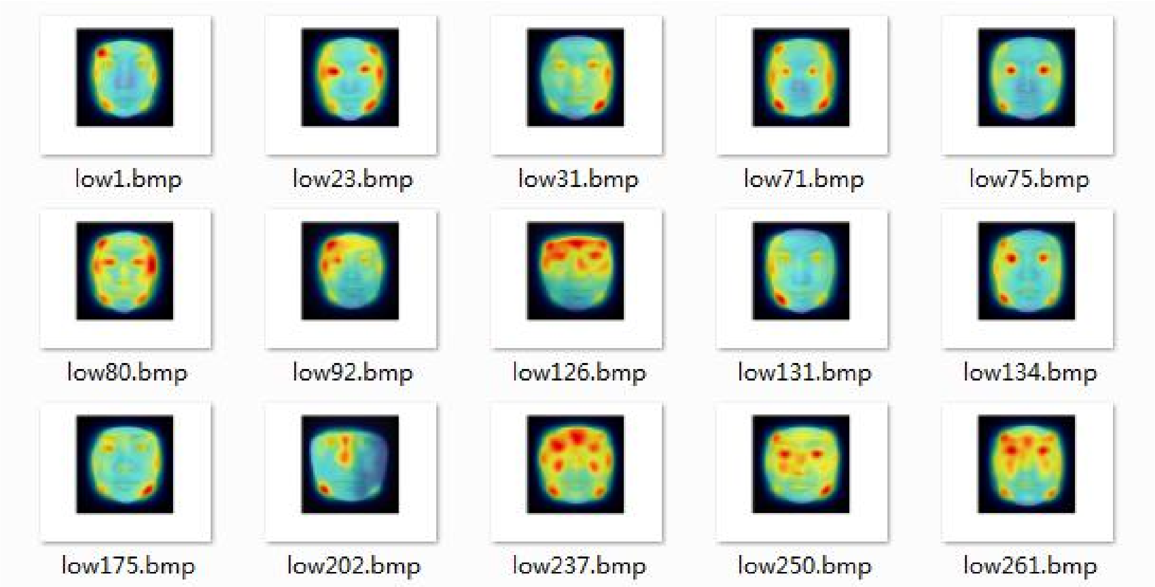

Thus, we can see that “nose” regions are not more salient than the other regions of face from both the high attractive group and the low attractive group.

It was added in the “Discussion” section, as follows:

Based on the the saliency literature (Santangelo, 2015), visual saliency have been checked to indicate that nose regions are not more salient than the other regions of face from both the high attractive faces and the low attractive faces.（On p. 16）

Once again, we would like to thank you for your careful readings of our manuscript. We have worked hard to address your concerns in our revised paper and look forward to hearing from you.

Sincerely yours,

The authors
